# Methionyl-tRNA synthetase overexpression is associated with poor clinical outcomes in non-small cell lung cancer

**DOI:** 10.1186/s12885-017-3452-9

**Published:** 2017-07-05

**Authors:** Eun Young Kim, Ji Ye Jung, Arum Kim, Kwangsoo Kim, Yoon Soo Chang

**Affiliations:** 1 Department of Internal Medicine, Yonsei University College of Medicine, Seoul, Republic of Korea; 2 Division of Clinical Bioinformatics, Biomedical Research Institute, Seoul National University Hospital, Seoul, Republic of Korea

**Keywords:** Aminoacyl-tRNA synthetase (ARS), Methionyl-tRNA synthetase (MRS), NSCLC, Lung cancer

## Abstract

**Background:**

Methionyl-tRNA synthetase (MRS) plays a critical role in initiating translation by transferring Met to the initiator tRNA (tRNA_i_^Met^) and protection against ROS-mediated damage, suggesting that its overexpression is related to cancer growth and drug resistance. In this study, the clinical implication of MRS expression in non-small cell lung cancer (NSCLC) was evaluated.

**Methods:**

Immunoblot and immunohistochemical (IHC) analyses were performed using tissue lysates and formalin-fixed paraffin embedded (FFPE) tissue blocks from wild type C57BL/6, LSL-Kras G12D, and LSL-Kras G12D:p53^fl/fl^ mice. For human studies, 12 paired adjacent normal appearing lung tissue lysates and cancer tissue lysates, in addition to 231 FFPE tissue samples, were used.

**Results:**

MRS was weakly expressed in the spleen and intestinal epithelium and only marginally expressed in the kidney, liver, and lungs of wild type C57BL/6 mice. On the other hand, MRS was strongly expressed in the neoplastic region of lung tissue from LSL-Kras G12D and LSL-Kras G12D:p53^fl/fl^ mice. Immunoblot analysis of the human normal appearing adjacent and lung cancer paired tissue lysates revealed cancer-specific MRS overexpression, which was related to mTORC1 activity. IHC analysis of the 231 FFPE lung cancer tissue samples showed that MRS expression was frequently detected in the cytoplasm of lung cancer cells (179 out of 231, 77.4%), with a small proportion (73 out of 231, 31.6%) also showing nuclear expression. The proportion of cases with positive MRS expression was higher in the advanced pStage subgroup (*P* = 0.018, χ^2^-test) and cases with MRS expression also had shorter DFS (161.6 vs 142.3, *P* = 0.014, log-rank test).

**Conclusions:**

Taken together, MRS is frequently overexpressed in NSCLC. Moreover, MRS is related to mTORC1 activity and its overexpression is associated with poor clinical outcomes, indicating that it has potential as a putative therapeutic target.

**Electronic supplementary material:**

The online version of this article (doi:10.1186/s12885-017-3452-9) contains supplementary material, which is available to authorized users.

## Background

Lung cancer is a major public health problem worldwide. This disease has a high prevalence and high mortality; specifically, lung cancer had the 3rd lowest survival rate among all cancers in 2014 [1, 2]. Thus, there is an urgent need for new diagnostic methods enabling early detection and for new treatment modalities for lung cancer.

Low-dose computerized tomography (LDCT) scans are currently used as a screening tool. Their use is supported by the National Lung Screening Trial (NLST), a randomized collected study involving more than 53,000 current or former heavy smokers [3]. However, lung cancer screening with LDCT has serious problems, such as false-positive rates exceeding 95%. This drawback leads to unnecessary repeated testing and increased costs [4]. Because of this and other limitations, new noninvasive methods for the early detection of lung cancer are needed. Multiple peripheral blood or body fluid matrix biomarkers in lung cancer have been proposed, such as protein biomolecules, miRNA, cell-free DNA, methylated DNA, circulating tumor cells, metabolites, and lipids [5]. However, protein markers are the only type of cancer biomarkers approved by the Food and Drug Administration (FDA) to date [6]. Protein biomarkers are easy to detect with high sensitivity and/or specificity in peripheral blood.

Aminoacyl-tRNA synthetases (ARSs) are housekeeping enzymes that catalyze the ligation of amino acids to their cognate transfer RNAs (tRNAs) with high fidelity [7]. Consuming one ATP in each reaction, these enzymes activate amino acids to aminoacyladenylates and then deliver the activated amino acids to the acceptor ends of tRNAs [8]. Mammalian ARSs have additional domains such as a glutathione S-transferase domain, a WHEP domain, leucine zipper domains, and α-helical appendices, which enable them to perform versatile intracellular and intercellular functions. Among the free-form ARSs, tryptophanyl-tRNA synthetase (WRS) and tyrosyl-tRNA synthetase (YRS) can be secreted and modified to control angiogenesis and immune responses in the tumor microenvironment. Truncation of the amino-terminal of WRS generates cytokines that suppress angiogenesis [9]. YRS is cleaved into N- and C-terminal domains, which have proangiogenic and immune activation functions, respectively [10, 11]. One the other hand, 8 different ARSs [bifunctional glutamyl-prolyl-tRNA synthetase (EPRS), isoleucyl-tRNA synthetase (IRS), leucyl-tRNA synthetase (LRS), glutaminyl-tRNA synthetase (QRS), lysyl-tRNA synthetase (KRS), arginyl-tRNA synthetase (RRS), aspartyl-tRNA synthetase (DRS), and methionyl tRNA synthetase (MRS)] form a complex with ARS-interacting multifunctional proteins (AIMPs) and play noncanonical roles [8]. EPRS is a translational silencer that suppresses vascular endothelial growth factor A [12]. KRS binds to microphthalmia-associated transcription factor (MITF) and is involved in the development of melanoma [13]. QRS interacts with apoptosis signal-regulating kinase 1 and suppresses apoptosis in a glutamine-dependent manner [14]. These reports suggested that ARS overexpression may impact cancer survival and progression and that ARS inhibitors are thus potential anticancer therapeutics. In addition, the multifunctional nature of ARSs and their localization to multiple areas suggest their potential as cancer diagnostic biomarkers in peripheral blood and tissue.

MRS is a critical enzyme in translation initiation and transfers Met to the initiator tRNA (tRNA_i_^Met^), suggesting that this enzyme may play an important role in tumor growth [15]. MRS increases ribosomal RNA biogenesis in the nucleolus and interacts with various signaling molecules such as mTORC1, GCN2, CDK4, and VEGFR [7, 16, 17]. After phosphorylation of MRS at Ser662 by UV-mediated DNA damage, MRS dissociates from AIMP3 and links the DNA damage responses to global translation control [15]. For this reason, MRS has been considered a strong biomarker candidate for the therapy of lung cancer.

Here we evaluated MRS expression in mouse tissue and in human lung cancer tissue to determine its clinical implications. In addition, we evaluated the relationship between MRS expression and the mTOR pathway, which plays a critical role in cancer growth and proliferation, to examine the relationship between tumor growth and MRS expression.

## Methods

### Study design and subjects

Immunoblot and immunohistochemical (IHC) analyses were performed using tissue lysates and paraffin-embedded tissue blocks from the major organs of 8-week-old wild type C57BL/6 mice. To evaluate MRS expression in mouse lung cancer tissue, LSL-Kras G12D and LSL-Kras G12D:p53^fl/fl^ mice were sacrificed 24 and 8 weeks after AdCre particle inhalation, respectively (https://ncifrederick.cancer.gov/Lasp/MouseRepository/Default.aspx). All animal work was approved by the Institutional Animal Care and Use Committee of Yonsei University (2014–0229-1) and followed the guidelines of the American Association for the Assessment and Accreditation of Laboratory Animal Care. To evaluate MRS expression in human lung cancer, 12 paired lysates from adjacent normal appearing lung tissue and cancer enriched tissue were analyzed by immunoblotting. Another set of 231 formalin-fixed paraffin embedded lung cancer tissue slides were analyzed by IHC. This study was approved by the IRB of Gangnam Severance Hospital (IRB #3–2014-0299) and was carried out in compliance with the Declaration of Helsinki (https://www.wma.net/policies-post/wma-declaration-of-helsinki-ethical-principles-for-medical-research-involving-human-subjects/##) and Korean GCP guidelines.

### Antibodies and immunoblotting

Anti-MRS and anti-LRS antibodies were purchased from Neomix Inc. (Suwon, Gyeonggi-do, Korea); anti-Ki67 antibodies were obtained from Abcam (Cambridge, UK). All other antibodies were obtained from Cell Signaling Technology (Danver, MA, USA) unless otherwise stated. Cells were harvested on ice and lysed in 2× Laemmli sample buffer containing protease and phosphatase inhibitors (GeneDepot, Barker, TX, USA). After sonication, 30–50 μg of lysate was separated by gel electrophoresis on 7.5 to 12% polyacrylamide gels and transferred onto nitrocellulose membranes (Bio-Rad Laboratories, Richmond, CA, USA). The expression level of each protein was quantified relative to that of β-actin.

### IHC analysis

The expression of MRS, Ki67, pS6, and pGSK-3β in non-small cell lung cancer (NSCLC) and mouse tissue samples was analyzed by IHC using the LABS^®^2 System (Dako, Carpinteria, CA, USA) according to the manufacturer’s instructions. Briefly, sections were deparaffinized, rehydrated, immersed in H_2_O_2_ methanol solution, and then incubated overnight with primary antibodies against MRS, Ki67, pS6, and pGSK-3β. Incubations were performed in antibody diluent (Dako) at dilutions of 1:500, 1:2000, 1:400, and 1:100, respectively. Sections were incubated for 10 min with a biotinylated linker and then processed using avidin/biotin IHC techniques. 3,3′-Diaminobenzidine (DAB) was used as a chromogen in conjunction with the Liquid DAB Substrate kit (Novacastra, UK). MRS expression was scored as the product of staining intensity and the percentage of positive cells. Staining intensity was classified as 0, 1, 2, or 3. Frequency was classified as 0 (<10%), 1 (10–50%), 2(51–80%), or 3 (>80%). Overexpression was defined as when the product of intensity and frequency was ≥2.

### Statistics

Clinically significant differences of MRS expression levels were identified using the χ^2^ test, Fisher’s exact test, and the independent 2 sample *t-*test. Disease free survival (DFS) was defined as the period from the time of surgery to the time of recurrence and overall survival (OS) was defined as the period from diagnosis to death. Predictive factors for DFS and OS were calculated using the Kaplan-Meier estimator and a Cox proportional hazards model. All significance tests were 2-tailed and *P*-values less than 0.05 were considered to indicate statistical significance. All analyses were performed using SPSS, version 20 (SPSS Inc., Chicago, IL, USA).

## Results

### MRS expression in various mouse tissues

**Fig. 1:**
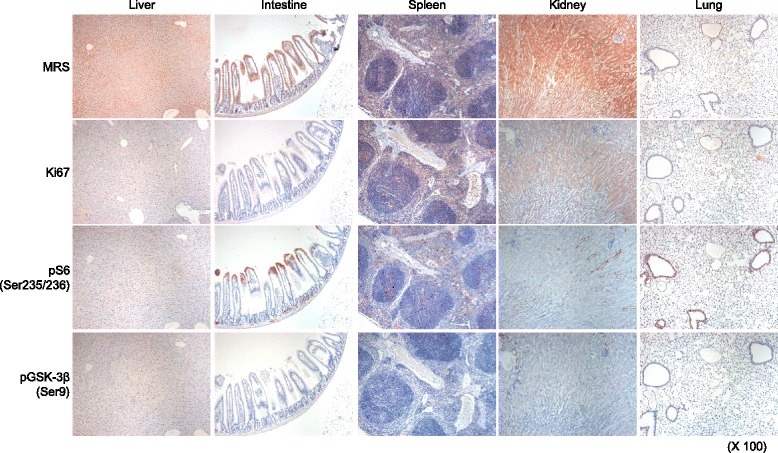
Expression of MRS, Ki67, and, mTOR signaling proteins in the wild type mouse organs. The expression of MRS, Ki67, pS6 (Ser235/236), and pGSK-3β (Ser9) was evaluated by IHC in tissue samples from 8-week-old wild type C57BL/6 mice. pS6 (Ser235/236) and pGSK-3β (Ser9) are surrogate markers of mTORC1 and mTORC2 activity, respectively. IHC analysis revealed that the enterocytes in the intestinal mucosal epithelium and the splenocytes in the periphery of the germinal center expressed MRS, whereas all other organs exhibited weak MRS expression. Photo was taken at low power magnification (X100)

To explore the potential of MRS as a therapeutic target of lung cancer, we first evaluated MRS expression in the non-neoplastic tissues of the major organs of 8-week-old wild type C57BL/6 mice (Fig. 1 and Additional file 1). Among the tested organs, MRS expression was highest in the spleen and intestinal epithelium, whereas MRS expression was weak in the kidney, liver, and lungs. Of the cell types in the intestine, enterocytes in the intestinal mucosal epithelium had strong cytoplasmic MRS expression. Splenocytes in the center of the periarterial lymphatic sheath did not express MRS, whereas those in the periphery of the germinal center did. In the kidney, the proximal convoluted tubule cells showed weak expression. With the exception of bronchial epithelial cells, which had weak MRS expression, the majority of lung cells did not express MRS. In the hepatic lobules, only hepatocytes had weak cytoplasmic MRS expression.

**Fig. 2:**
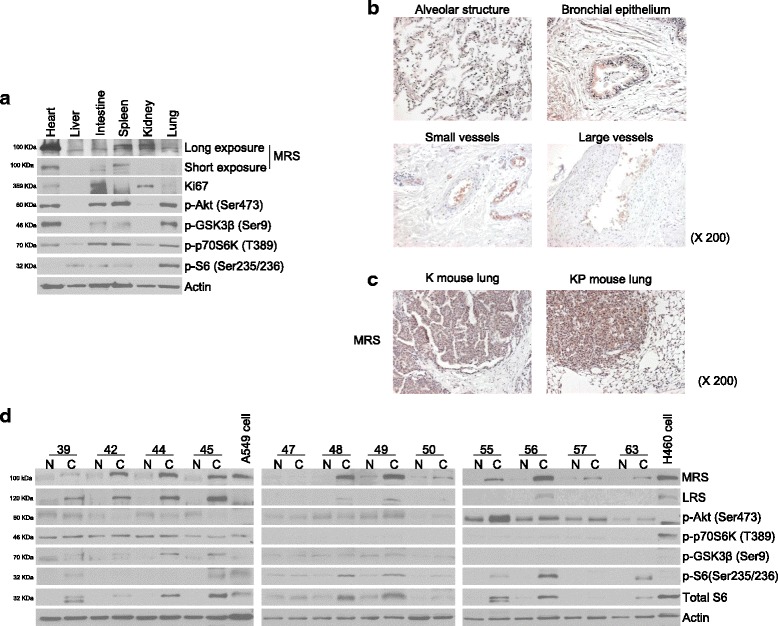
Expression of MRS, Ki67, and, mTOR signaling proteins in the wild type mouse organs (**a**) and MRS expression in lung cancer and normal appearing adjacent lung tissue specimens (**b**-**d**). **a** Expression of MRS, mTOR signaling proteins, and Ki67 was evaluated by immunoblotting using tissue lysates from 8-week-old wild type C57BL/6 mice. IHC of MRS expression in (**b**) human adjacent normal lung tissue and (**c**) LSL-Kras G12D and LSL-Kras-G12D:p53^fl/fl^ murine lung cancer tissue. **d** Immunoblot analysis of MRS expression and mTOR components in 12 pairs of lung cancer and adjacent normal appearing lung tissue lysates

MRS plays a critical role in the initial step of protein synthesis and is also involved in signaling via its interactions with mTORC1 and CDK4 [7, 16, 17]. Because MRS also suggested to be involved in the cell cycle progression via increasing stability of CDK4 (unpublished data)., we next investigated whether MRS expression is related cell proliferation (Fig. 1, Additional files 1 and 2 A-B) and/or to mTOR activity (Fig. 1, Additional files 1 and 2 C-F) [15]. Ki67 was strongly expressed in the spleen and intestine, two organs exhibiting high MRS expression. The kidney was an exception to this trend in that it did not exhibit expression of MRS or mTOR markers, but did show Ki67 expression. We performed IHC of renal tissue to validate this finding and observed that Ki67 was occasionally detected in the nuclei of proximal convoluted tubule cells. To evaluate the relationship between MRS expression and mTOR activity on the cellular level, the expression levels of pS6 (Ser235/236) and pGSK-3β (Ser 9), surrogate markers of mTORC1 and mTORC2, respectively, were evaluated in the same mouse tissue samples by IHC. pS6 (Ser235/236) was strongly expressed in intestinal and bronchial epithelial cells, with expression foci also observed in splenocytes, renal tubular cells, and hepatocytes. pGSK-3β (Ser 9), an mTORC2 marker, showed a similar but weaker expression than that of pS6, indicating that the expression patterns of mTOR surrogate markers and MRS correspond with each other in these non-neoplastic mouse tissues. To validate MRS expression in the tested organs, we performed immunoblot analysis of the tissue lysates and included a heart lysate as a control, which was shown to have high MRS expression in previous experiments (Fig. 2a). Similar to the IHC results, MRS expression intensity was highest in the spleen, followed by the intestine and then the other organs.

### MRS is frequently and specifically overexpressed in lung cancer and is related to mTORC1 activity

To determine if MRS expression is specific to lung cancer, MRS expression was then evaluated in normal appearing adjacent lung tissue (Fig. 2b). MRS was not detected in alveolar type I and type II pneumocytes, nor was it detected in the vascular structures. However, MRS was weakly expressed in epithelial cells in the bronchioles and bronchi. Lung cancer-specific MRS overexpression was observed in Kras-LSL G12D and Kras-LSL G12D:p53^fl/fl^ murine lung cancer cells (Fig. 2c). In this model, lung cancer is induced by AdCre inhalation. Lung cancer-specific MRS overexpression was further evaluated by immunoblot analysis of lysates from human lung cancer and adjacent normal appearing lung tissue (Table 1 and Fig. 2d). Of the 12 pairs of cancer and normal tissue lysates, 9 pairs (75%) showed clear overexpression of MRS in the lung cancer tissue lysates. No pair exhibited higher MRS expression in the normal appearing lung tissue lysate compared with the cancer lysate.

**Table 1:** Clinical and pathological characteristics of the samples used for immunoblotting

Random No.	Age range	Cell type	Differentiation	Smoking	TNM	Stage
A39	70s	Adeno	n.s.^a^	None smoker	T3N0M0	IIB
A42	40s	Adeno	acinar type	None smoker	T1aN2M0	IIIA
A44	70s	Adeno	solid predominant	Ex-smoker	T2aN0M0	IB
A45	70s	Adeno	papillary predominant	None smoker	T1bN0M0	IA
A47	60s	Squamous	n.s^a^	None smoker	T2aN0M0	IB
A48	60s	Squamous	moderately differentiated	Current smoker	T2aN1M0	IIA
A49	60s	Squamous	poorly differentiated	Ex-smoker	T2aN0M0	IB
A50	60s	Adeno	micropapillary predominant pattern	Ex-smoker	T2aN1M0	IIA
A55	50s	Adeno	micropapillary predominant with extracellur mucin fomation	Current smoker	T2aN2M0	IIIA
A56	70s	Adeno	acinar predominant	Current smoker	T1bN2M0	IIIA
A57	60s	Squamous	moderately differentiated	Ex-smoker	T2bN2M0	IIIA
A63	70s	Adeno	acinar predominant	None smoker	T2aN0M0	IB

n.s.^a^: not specified

To identify the molecules associated with MRS overexpression, we analyzed the expression of mTOR signaling molecules (Fig. 2d). Among the ARSs tested, LRS showed a similar expression pattern to MRS, although MRS was more frequently detected than LRS. This finding is interesting because LRS detects intracellular leucine and activates mTORC1 signaling [7]. Interestingly, immunoblot analysis of tumor tissue lysates revealed that MRS expression was more closely associated with mTORC1, p70S6K, and pS6 expression as opposed to mTORC2 expression. To make the relationship between MRS and mTOR1 signaling clearer, serial sections from a number of human NSCLC was immunostained and analyzed for their correlation (Additional file 3). The expression of MRS showed a significant positive correlation with expression of pS6, LRS, and HSP70. These findings are consistent with the immunoblot analyses of wild type mouse tissue lysates. Taken together, our data indicate that MRS is frequently expressed and is highly specific to NSCLC, suggesting that MRS is one of potential candidate of therapeutic target of lung cancer.

### MRS expression is related to poor prognosis in NSCLC

**Fig. 3:**
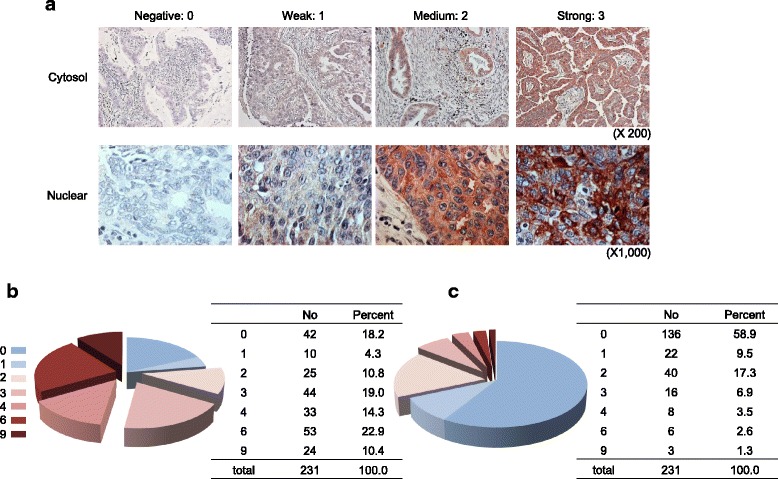
Frequency and intensity of MRS expression in NSCLC tissue. **a** Representative images of cytoplasmic and nuclear MRS expression in NSCLC tissue. Distribution of cytoplasmic (**b**) and nuclear (**c**) expression of MRS in 231 NSCLC cases as assessed by IHC. The expression score was calculated as the product of the intensity and the frequency

To further validate the clinical implications of MRS expression in lung cancer, we assessed MRS expression in 231 paraffin-embedded NSCLC tissue specimens. MRS was mainly expressed in the cytoplasm of lung cancer cells (Fig. 3a and b). MRS expression was scored as follows: product of intensity and frequency 0–1, negative/trace expression; product of intensity and frequency more than 2, positive MRS expression. A total of 179 (77.4%) NSCLC cases showed positive MRS cytoplasmic expression. On the other hands, 73 (31.6%) cases showed nuclear expression. The cytoplasmic MRS-positive expression rate in NSCLCs was comparable to the immunoblotting results.

To explore the clinical implications of MRS expression in NSCLC, cases were classified into the following 2 groups according to cytoplasmic MRS expression status: negative/trace (score 0–1) and positive expression (score 2–9). The clinical/pathological characteristics of the two groups were then compared (Table 2). A total of 78 (33.8%) out of the 231 cases were female; the mean patient age was 61.6 ± 10.73 years. These parameters are similar to the general characteristics of NSCLC. Among the tested parameters, the proportion of MRS-positive cases gradually increased as the pStage increased (*p* = 0.018, Pearson’s χ^2^-test). Age, sex, smoking history, pathologic classification, and stage did not differ between the MRS expression groups.

**Table 2:** Clinical and pathological characteristics of the patients according to the expression of MRS

		Expression of MRS		*P*-value
		Negative/Trace (*n* = 52)	Positive (*n* = 179)	
Age (years)		61.1 ± 10.79	62.0 ± 10.71	0.613
Gender				
	Male	33	120	0.631
	Female	19	59	
Diameter (cm)		3.5 ± 1.96	3.7 ± 1.93	0.602
Smoking status	None-smoker	22	63	0.762
	Ex-smoker	11	38	
	Current smoker	15	63	
	Unknown	4	12	
Pack Year		24.1 ± 25.40	25.1 ± 24.98	0.102
Pathology				
	Adenocarcinoma	31	99	0.597
	Squamous cancer	21	74	
	Others^a^	0	6	
PET-SUV		8.6 ± 4.41	9.1 ± 5.96	0.868
pStage				
	I	38	94	0.018
	II	10	34	
	III	4	48	
	IV	0	2	

^a^These cases were composed of 2 large cell carcinomas, 2 atypical carcinoids, 1 mucoepidermoid carcinoma, and 1 pleomorphic carcinoma

**Fig. 4:**
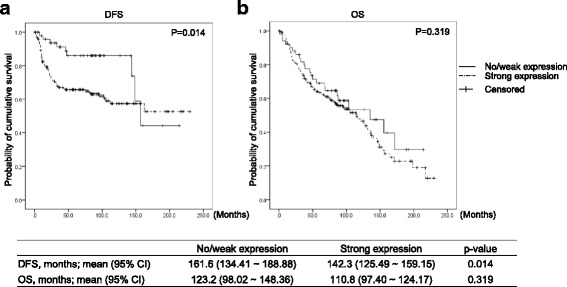
Impact of cytoplasmic MRS expression on (**a**) DFS and (**b**) OS in 231 NSCLC cases. *P*-values were obtained by the log-rank test

To evaluate the effect of MRS expression on clinical outcomes, disease-free survival (DFS) and overall survival (OS) of the groups were compared using Kaplan-Myer estimators (Fig. 4). The median follow-up duration was 121.4 months (95% CI; 100.34–142.46 months); 78 (32.1%) cases experienced lung cancer recurrence. A total of 124 (51.0%) patients died during the follow-up period. The MRS expression group showed poor clinical outcomes, in particular significantly poor DFS (161.6 vs 142.3 months, *P* = 0.014, log-rank test). The OS curves of the 2 groups were clearly separated, with the OS curve of the MRS expression group shorter than that of the other group; however, this trend did not reach statistical significance.

To test the hypothesis that MRS overexpression is an independent prognostic factor in NSCLC, univariate and multivariate analyses were performed using a Cox regression hazard model (Table 3). Univariate analysis revealed that MRS expression and pStage were significant predictors of poor DFS. Multivariate analysis including MRS expression, age, sex, smoking status, pStage, and pathology showed that pStage was the only factor that significantly influenced DFS.

**Table 3:** Univariate and multivariate analyses for DFS

		Univariate analysis			Multivariate analysis		
Variables		HR	95% CI	*P*-value	HR	95% CI	*P*-value
Age	< 65	1	reference	0.969	1	reference	0.722
	≥ 65	0.991	0.628∼1.564		1.092	0.672 ~ 1.775	
Sex	Male	1	reference	0.060	1	reference	0.055
	Female	0.608	0.362 ~ 1.021		0.460	0.208 ~ 1.018	
Smoking status	None	1	reference	0.106	1	reference	0.586
	Ex-smoker	2.112	1.164∼3.831		1.250	0.560 ~ 2.794	
	Current smoker	1.35	0.775∼2.353		0.720	0.328 ~ 1.580	
MRS overexpression	Negative/Trace	1	reference	0.017	1	reference	0.064
	Positive	2.33	1.161 ~ 4.677		2.017	0.985 ~ 4.130	
pStage	I	1	reference	<0.001	1	reference	< 0.001
	II	2.412	1.393∼4.175		2.547	1.349 ~ 4.807	
	III	3.141	1.817–5.429		2.744	1.537 ~ 4.899	
	IV	32.208	7.206–143.957		26.047	5.192 ~ 130.68	
Histologic differentiation	Well	1	reference	0.415	1	reference	0.687
	Moderate	1.767	0.904–3.454		1.150	0.555 ~ 2.383	
	Poor	1.628	0.734–3.034		1.003	0.450 ~ 2.235	

## Discussion

Besides its canonical role as a translation initiator by transferring Met to initiator tRNA_i_^Met^, MRS has multiple noncanonical functions. MRS senses intracellular Met, leading to the activation of mTORC1 signaling [18], and also stabilizes CDK4, thereby inducing cell cycle progression (unpublished data). MRS also detects intracellular oxidative stress, defends cells from DNA damage, and controls protein synthesis [15]. In addition to these roles, the discovery of MRS genetic variations in the late-onset autosomal dominant Charcot–Marie–Tooth neuropathy indicates that MRS has additional uncharacterized roles [19].

We evaluated MRS expression in the organs of wild type C57BL/6 mice and observed only low levels of expression. Remarkably, MRS was strongly expressed in heart tissue, whereas MRS expression was lower in hepatic tissues, where protein is more actively synthesized than in the spleen and intestine. MRS expression was weakly correlated with mTOR signaling components in wild type mouse tissue samples; however, the correlation between mTORC1 signaling and MRS overexpression was more prominent in the NSCLC tissue samples. These findings suggest that the regulatory mechanisms are working properly in normal tissue, but that in cancer tissue these mechanisms are dysregulated and the cancer cells are dependent on mTOR signaling. However, more evidence is required to prove that activation of the mTOR pathway induces MRS overexpression.

The majority of the NSCLC tissue samples showed clear expression of MRS, with cytoplasmic expression more frequent than nuclear expression. Nuclear MRS has unique functions in growth stimulating conditions related to ribosomal RNA biosynthesis [20]. In this study, a small percentage (31.6%) of NSCLC cells showed nuclear MRS expression, which was not statistically significant. This finding might be due to the fact that few cases exhibited nuclear MRS overexpression, meaning that the sample size was too small to detect significance.

When the Kaplan-Myer estimator was used to assess the clinical implications of MRS expression, MRS expression was not found to be an independent prognostic factor for DFS. This finding may be due to the positive correlation between the degree of MRS expression and the age of the study participant (Pearson correlation coefficient = 0.145, *P* = 0.027) and the strong relationship between pStage and MRS expression. Evaluation of the individual TNM staging components revealed that maximal tumor diameter and T staging component were associated with MRS expression. Moreover, MRS expression was significantly higher with each N stage increment (*P* = 0.007, Pearson’s χ^2^-test), suggesting that MRS may play a role in lymphangitic metastasis. Using the gene expression datasets of lung adenocarcinoma and lung squamous cell carcinoma from the TCGA, the effect of MRS gene expression level on clinical outcome was analyzed. In the lung cancer dataset that includes all stages, there were no prominent differences in clinical outcome according to MRS level, whereas in the subgroup of stage III ~ IV the patients with elevated MRS level tended to show a poor clinical outcome (Additional file 4 A-C).

We anticipate that this initial confirmation of MRS expression in cancer tissue and demonstration of its clinical implications will lead into future investigations of the potential of MRS expression a therapeutic target for drug development. To be developed as a diagnostic biomarker, MRS expression needs to be easily evaluated in readily obtainable bodily fluid. Also, the mechanisms driving MRS overexpression need to be elucidated. One possible explanation of MRS overexpression is copy number gain at the MRS gene locus (COSMIC); however, only limited cases showed copy number gain. In order to develop MRS as a therapeutic target, a targetable site also needs to be identified in MRS.

## Conclusions

In conclusion, MRS is specifically and frequently overexpressed in NSCLC tissue. MRS overexpression in NSCLC tissue was related to mTORC1 activity and poor clinical outcomes, suggesting that further studies on MRS overexpression are warranted to pursue MRS as a therapeutic target.

## Additional files

Additional file 1: Supporting data 1.pptx. Expression of MRS, Ki67, and, mTOR signaling proteins in the wild type mouse organs. The expression of MRS, Ki67, pS6 (Ser235/236), and pGSK-3β (Ser9) was evaluated by IHC in tissue samples from 8-week-old wild type C57BL/6 mice. Photo was taken at high power magnification (X1000). (PPTX 6359 kb)

Additional file 2: Supporting data 2.pptx Double immunofluorescent staining of MRS with Ki67, and, mTOR signaling proteins in the wild type mouse organs. The expression of MRS with Ki67 (A, B), pS6 (Ser235/236) (C, D), and pGSK-3β (Ser9) (E, F) was evaluated by double immunofluorescent staining in tissue samples from 8-week-old wild type C57BL/6 mice. (MRS: red, others: green). Note that A, C, E was low power magnification (X 100) and B, D, F are high power magnification (X 1000). (PPTX 41087 kb)

Additional file 3: Supporting data 3.pptx Expression of MRS and other related proteins in human NSCLC (A) and their correlation Table (B). (A) The expression of proteins was evaluated by IHC in tissue samples from the same section. (B) Pearson correlation coefficient table relative to the expression of MRS expression. *P*-value was obtained from bivariate correlation analysis. (PPTX 2398 kb)

Additional file 4: Supporting data 4.pptx Survival analysis according to the MRS level from TCGA NSCLC data set. Lung cancer cases of (A) all stages, (B) stage I ~ II, and (C) and III ~ IV were selected from lung adenocarcinoma and lung squamous cell carcinoma TCGA data set and DFS and OS was evaluated according to the MRS level. The expression of MRS was divided into two groups, upper 50% and lower 50%, based on the median value. P- values was obtained from Log-rank test. (PPTX 892 kb)

## Abbreviations

AIMP: ARS-interacting multifunctional protein
ARS: Aminoacyl-tRNA synthetase
DFS: Disease free survival
FFPE: Formalin-fixed paraffin embedded
IHC: Immunohistochemistry
MRS: Methionyl-tRNA synthetase
NSCLC: Non-small cell lung cancer
OS: Overall survival
PET-SUVs: Positron Emission Tomography-Standardized Uptake Values
