# Supplementary material for: Methionyl-tRNA synthetase overexpression is associated with poor clinical outcomes in non-small cell lung cancer
Source: BMC Cancer. 2017 Jul 5;17:467. doi: 10.1186/s12885-017-3452-9 (PMC5497355; doi:10.1186/s12885-017-3452-9)
Supplement: Supplementary file 1 — Supporting data 1.pptx. Expression of MRS, Ki67, and, mTOR signaling proteins in the wild type mouse organs. The expression of MRS, Ki67, pS6 (Ser235/236), and pGSK-3β (Ser9) was evaluated by IHC in tissue samples from 8-week-old wild type C57BL/6 mice. Photo was taken at high power magnification (X1000). (PPTX 6359 kb) [file 12885_2017_3452_MOESM1_ESM.pptx]

## Slide 1
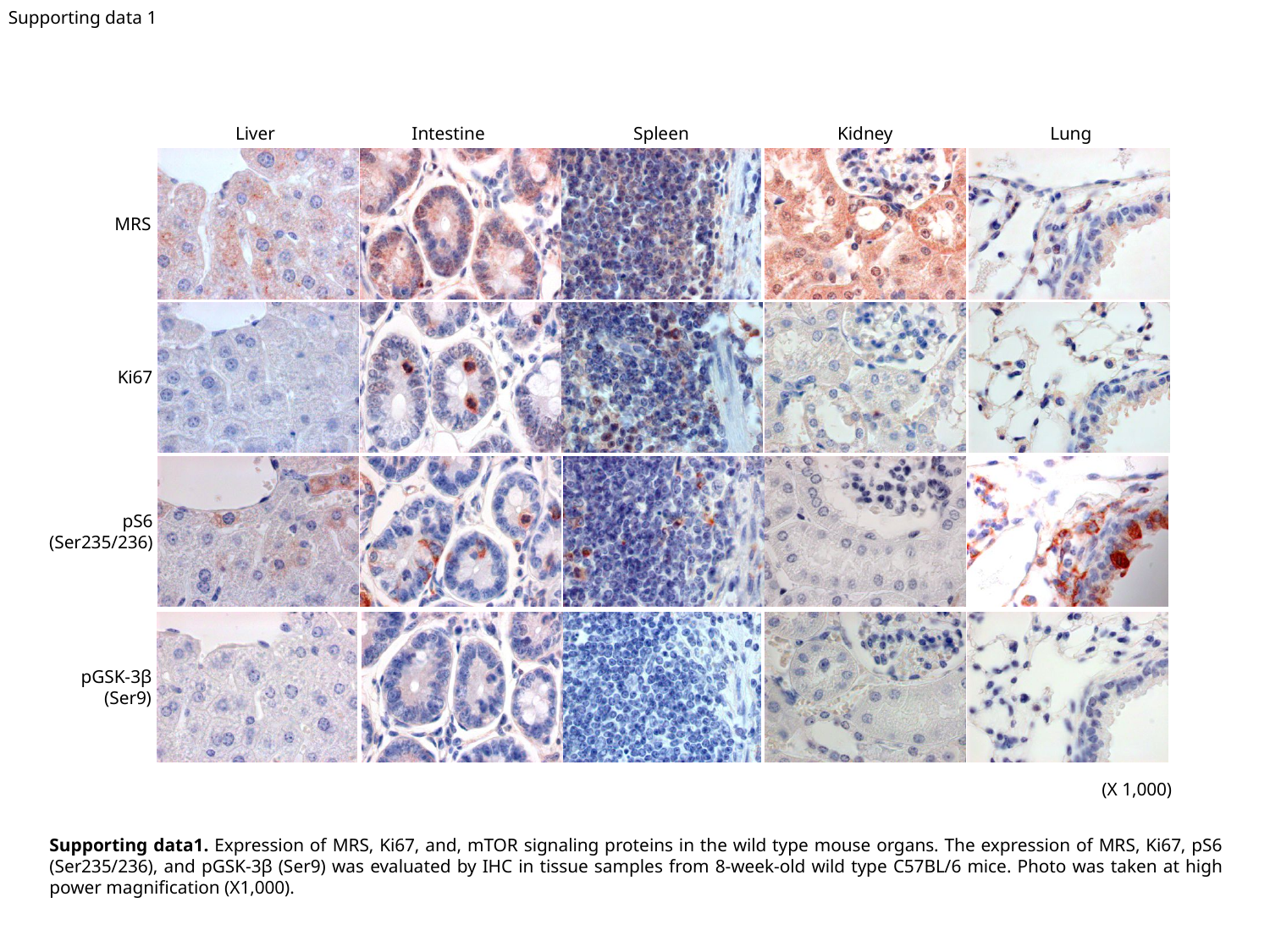

Supporting data 1
Liver
Intestine
Spleen
Kidney
Lung
MRS
Ki67
pS6
(Ser235/236)
pGSK-3β
(Ser9)
(X 1,000)
Supporting data1. Expression of MRS, Ki67, and, mTOR signaling proteins in the wild type mouse organs. The expression of MRS, Ki67, pS6 (Ser235/236), and pGSK-3β (Ser9) was evaluated by IHC in tissue samples from 8-week-old wild type C57BL/6 mice. Photo was taken at high power magnification (X1,000).
