# Supplementary material for: Methionyl-tRNA synthetase overexpression is associated with poor clinical outcomes in non-small cell lung cancer
Source: BMC Cancer. 2017 Jul 5;17:467. doi: 10.1186/s12885-017-3452-9 (PMC5497355; doi:10.1186/s12885-017-3452-9)
Supplement: Supplementary file 2 — Supporting data 2.pptx Double immunofluorescent staining of MRS with Ki67, and, mTOR signaling proteins in the wild type mouse organs. The expression of MRS with Ki67 (A, B), pS6 (Ser235/236) (C, D), and pGSK-3β (Ser9) (E, F) was evaluated by double immunofluorescent staining in tissue samples from 8-week-old wild type C57BL/6 mice. (MRS: red, others: green). Note that A, C, E was low power magnification (X 100) and B, D, F are high power magnification (X 1000). (PPTX 41087 kb) [file 12885_2017_3452_MOESM2_ESM.pptx]

## Slide 1
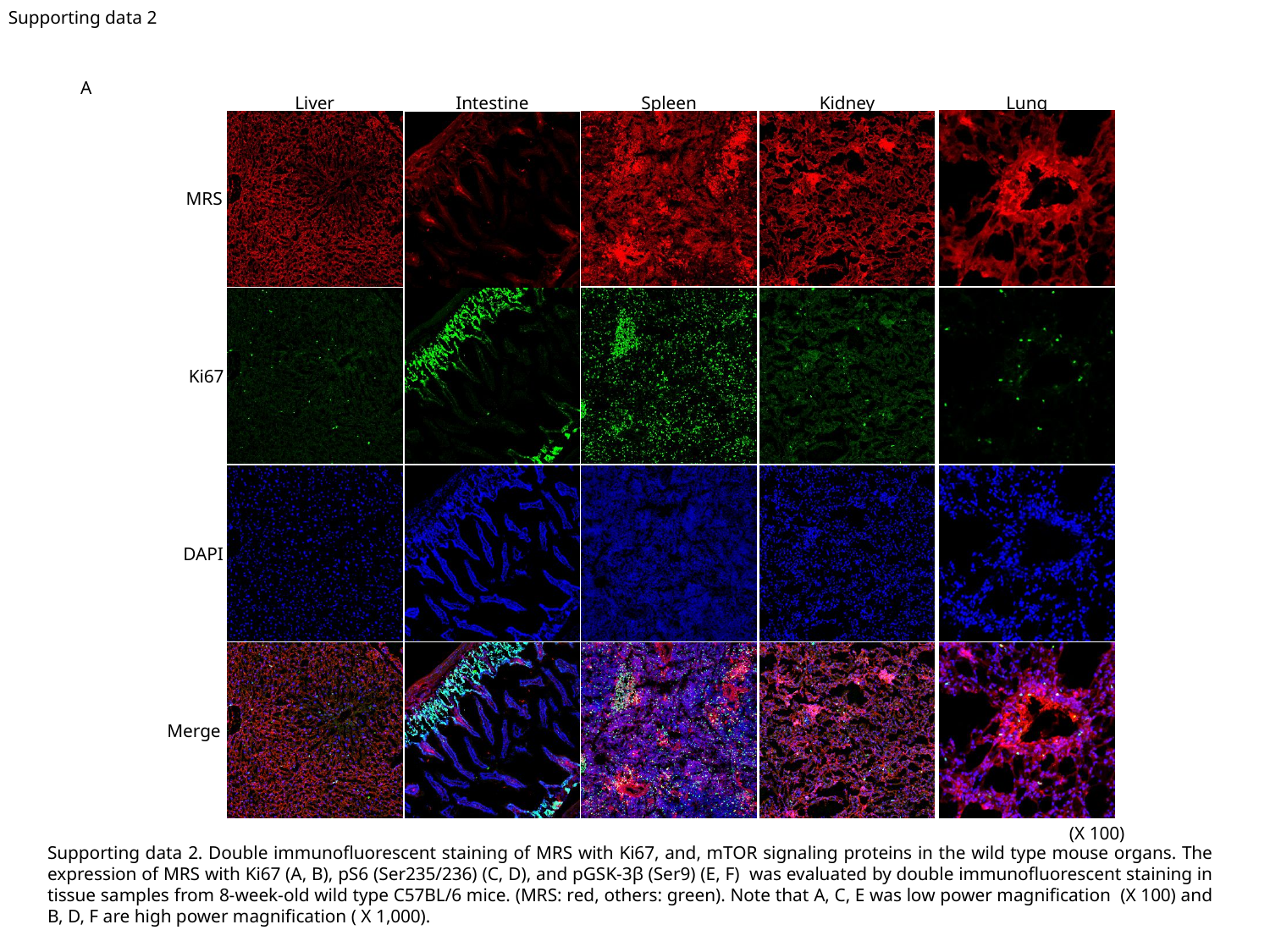

Supporting data 2
A
Liver
Intestine
Spleen
Kidney
Lung
MRS
Ki67
DAPI
Merge
(X 100)
Supporting data 2. Double immunofluorescent staining of MRS with Ki67, and, mTOR signaling proteins in the wild type mouse organs. The expression of MRS with Ki67 (A, B), pS6 (Ser235/236) (C, D), and pGSK-3β (Ser9) (E, F) was evaluated by double immunofluorescent staining in tissue samples from 8-week-old wild type C57BL/6 mice. (MRS: red, others: green). Note that A, C, E was low power magnification (X 100) and B, D, F are high power magnification ( X 1,000).

## Slide 2
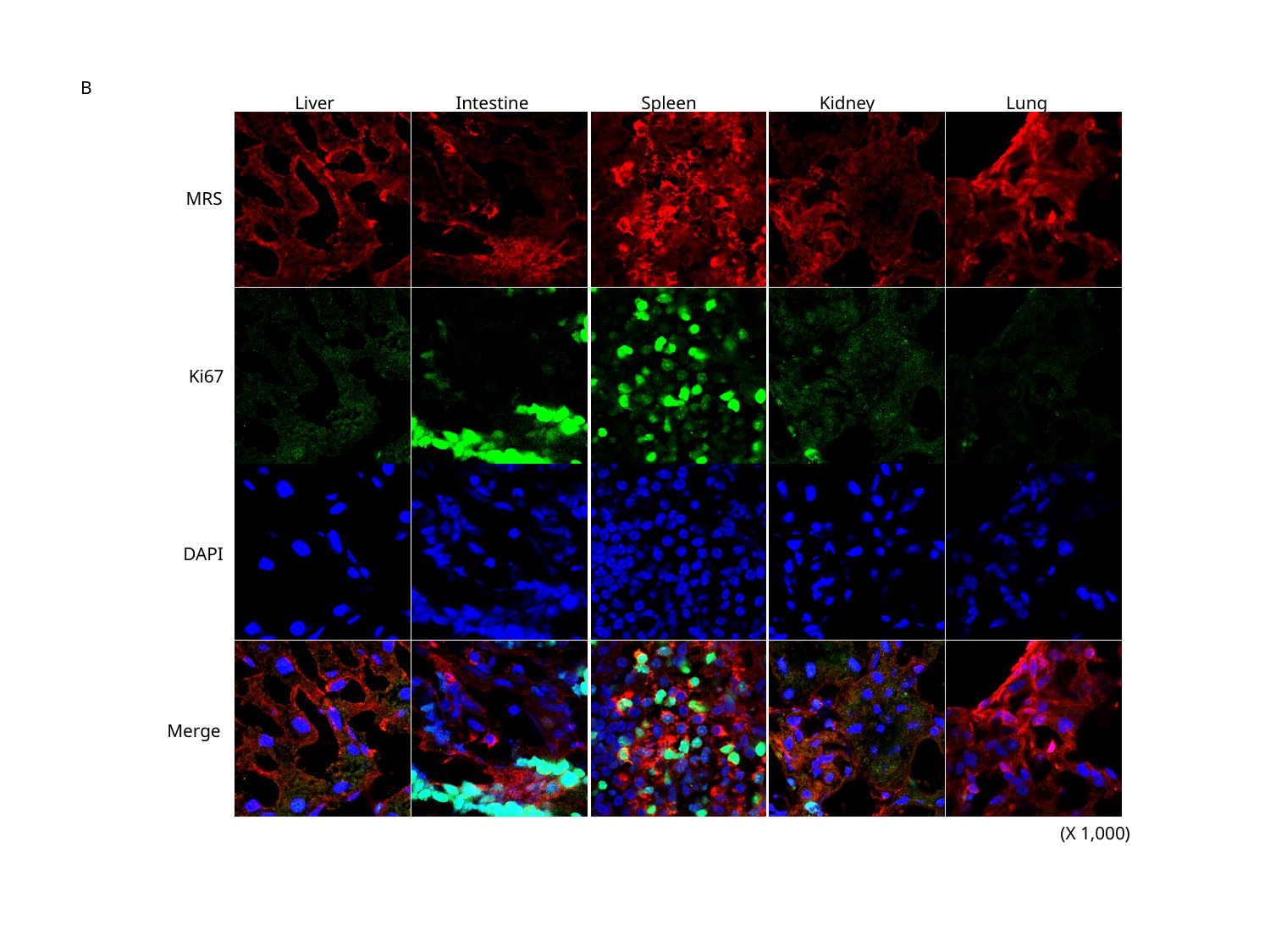

B
Liver
Intestine
Spleen
Kidney
Lung
MRS
Ki67
DAPI
Merge
(X 1,000)

## Slide 3
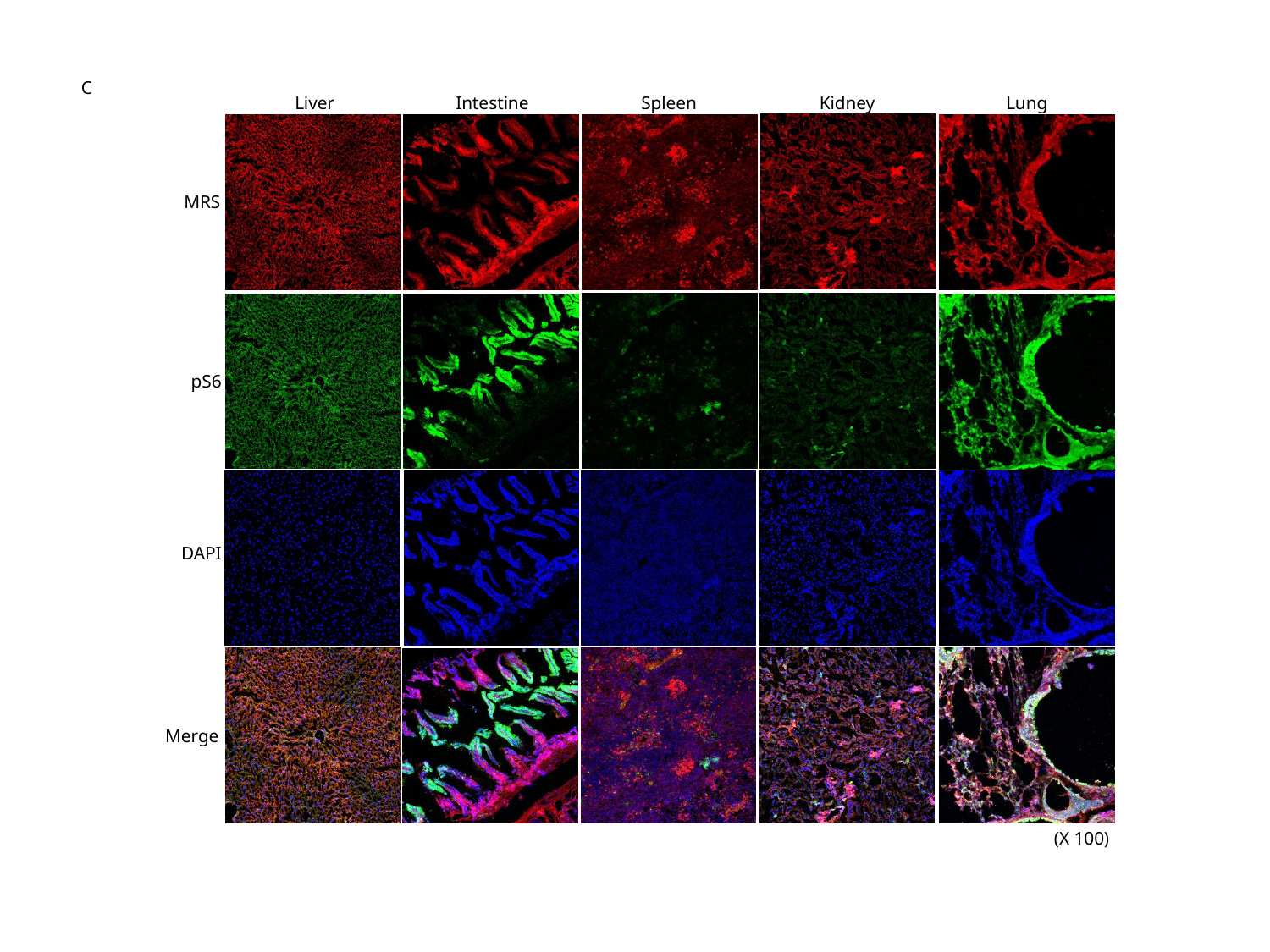

C
Liver
Intestine
Spleen
Kidney
Lung
MRS
pS6
DAPI
Merge
(X 100)

## Slide 4
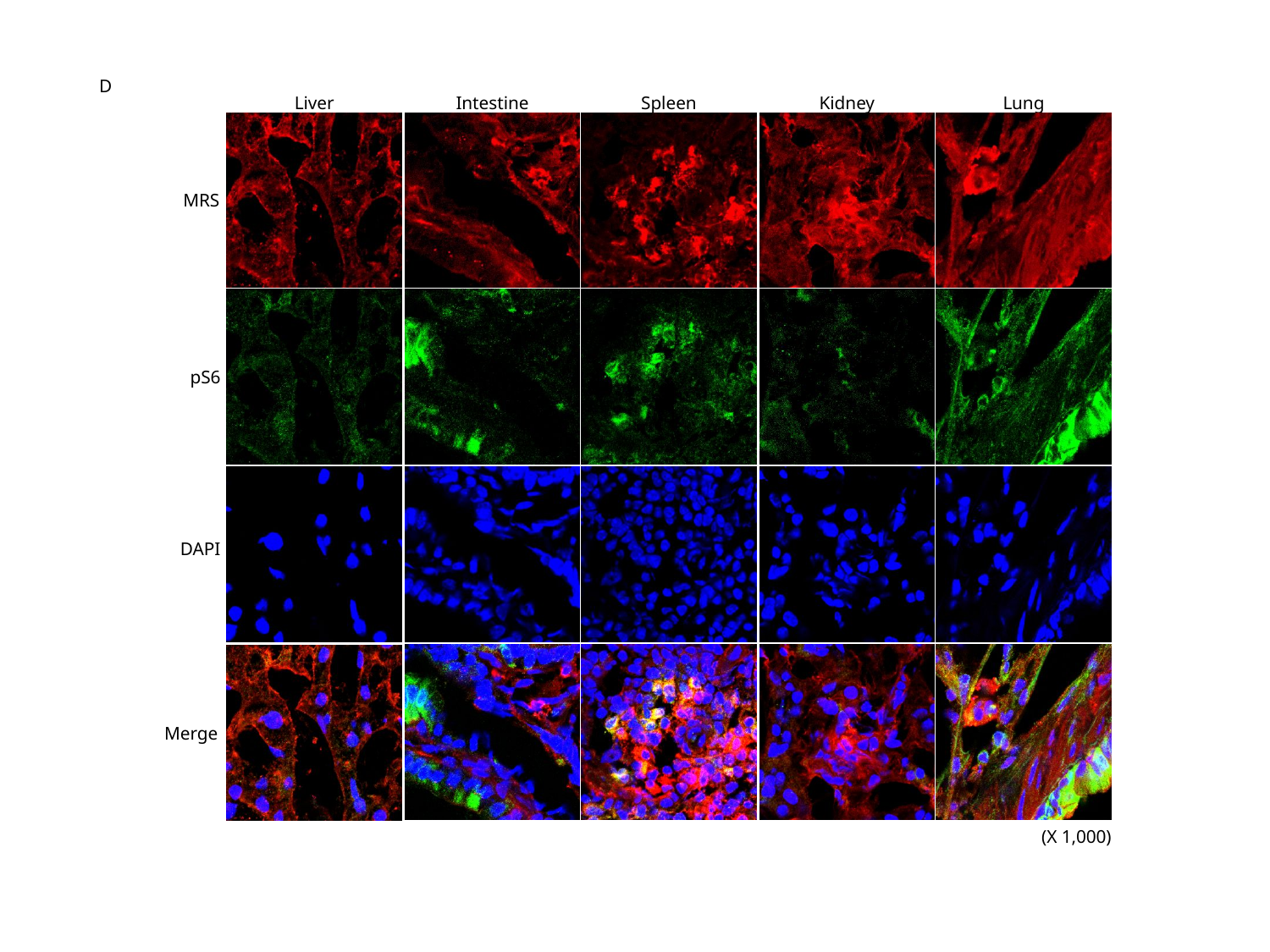

D
Liver
Intestine
Spleen
Kidney
Lung
MRS
pS6
DAPI
Merge
(X 1,000)

## Slide 5
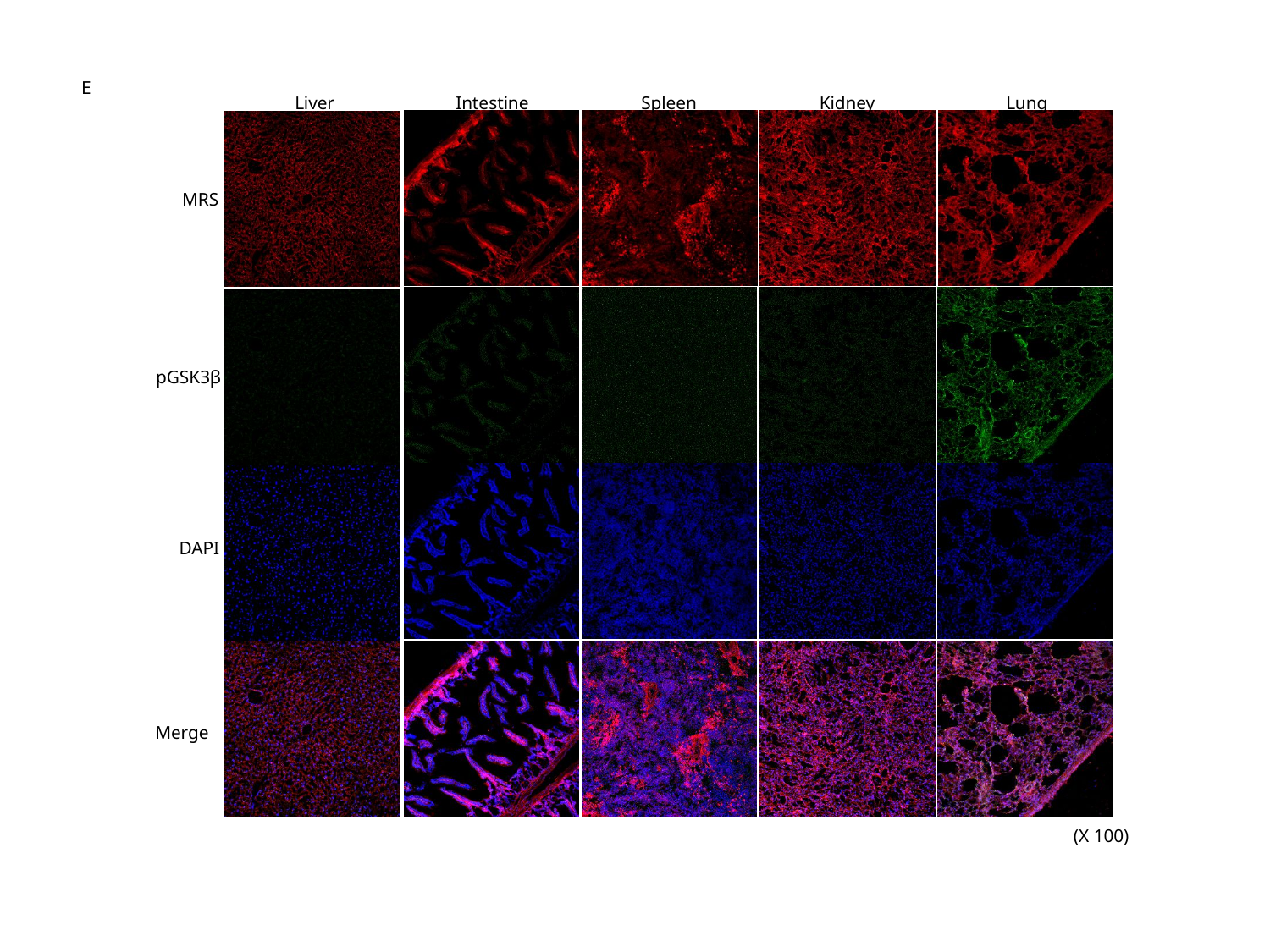

E
Liver
Intestine
Spleen
Kidney
Lung
MRS
pGSK3β
DAPI
Merge
(X 100)

## Slide 6
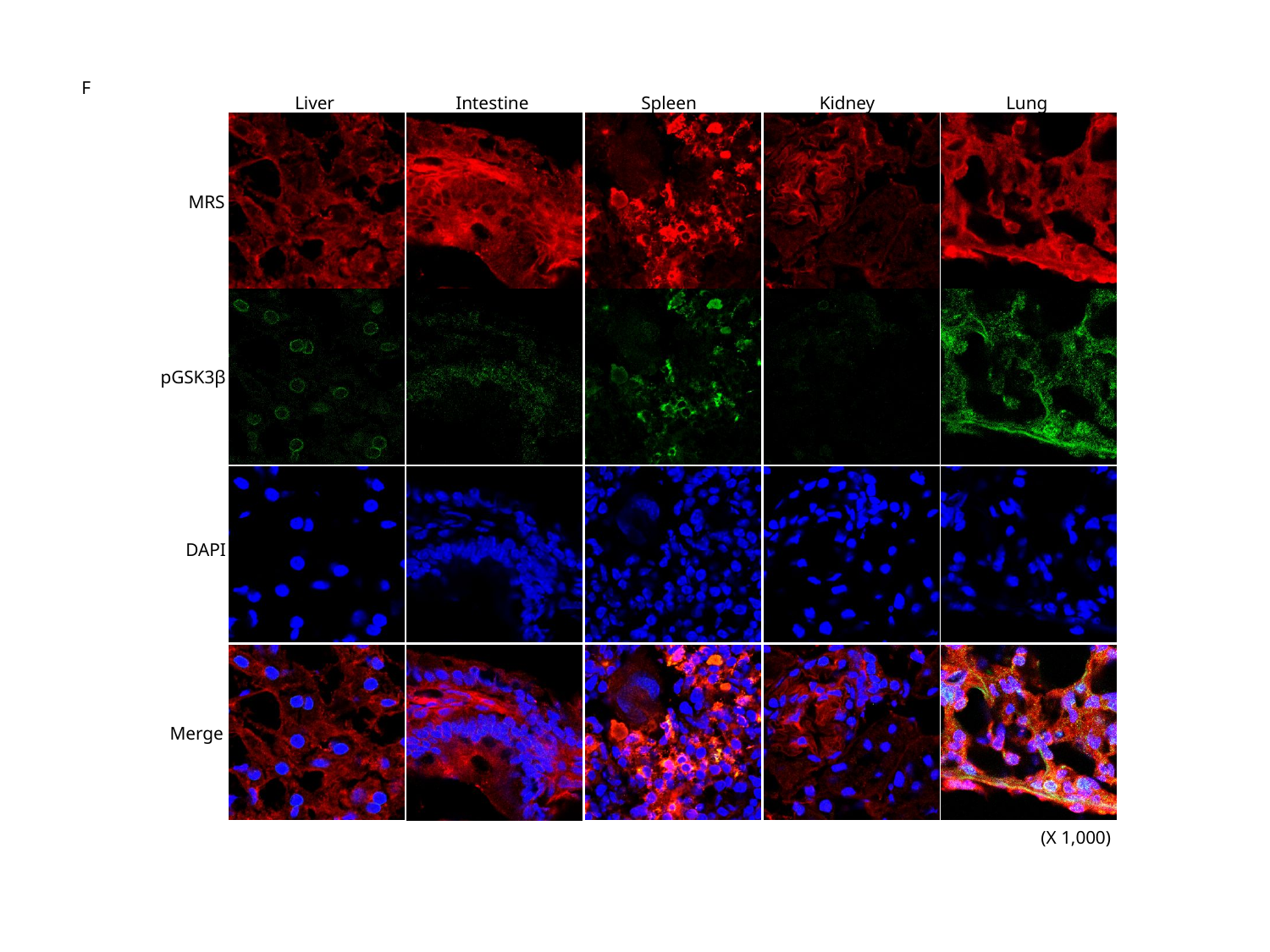

F
Liver
Intestine
Spleen
Kidney
Lung
MRS
pGSK3β
DAPI
Merge
(X 1,000)
