# Supplementary material for: Methionyl-tRNA synthetase overexpression is associated with poor clinical outcomes in non-small cell lung cancer
Source: BMC Cancer. 2017 Jul 5;17:467. doi: 10.1186/s12885-017-3452-9 (PMC5497355; doi:10.1186/s12885-017-3452-9)
Supplement: Supplementary file 3 — Supporting data 3.pptx Expression of MRS and other related proteins in human NSCLC (A) and their correlation Table (B). (A) The expression of proteins was evaluated by IHC in tissue samples from the same section. (B) Pearson correlation coefficient table relative to the expression of MRS expression. P-value was obtained from bivariate correlation analysis. (PPTX 2398 kb) [file 12885_2017_3452_MOESM3_ESM.pptx]

## Slide 1
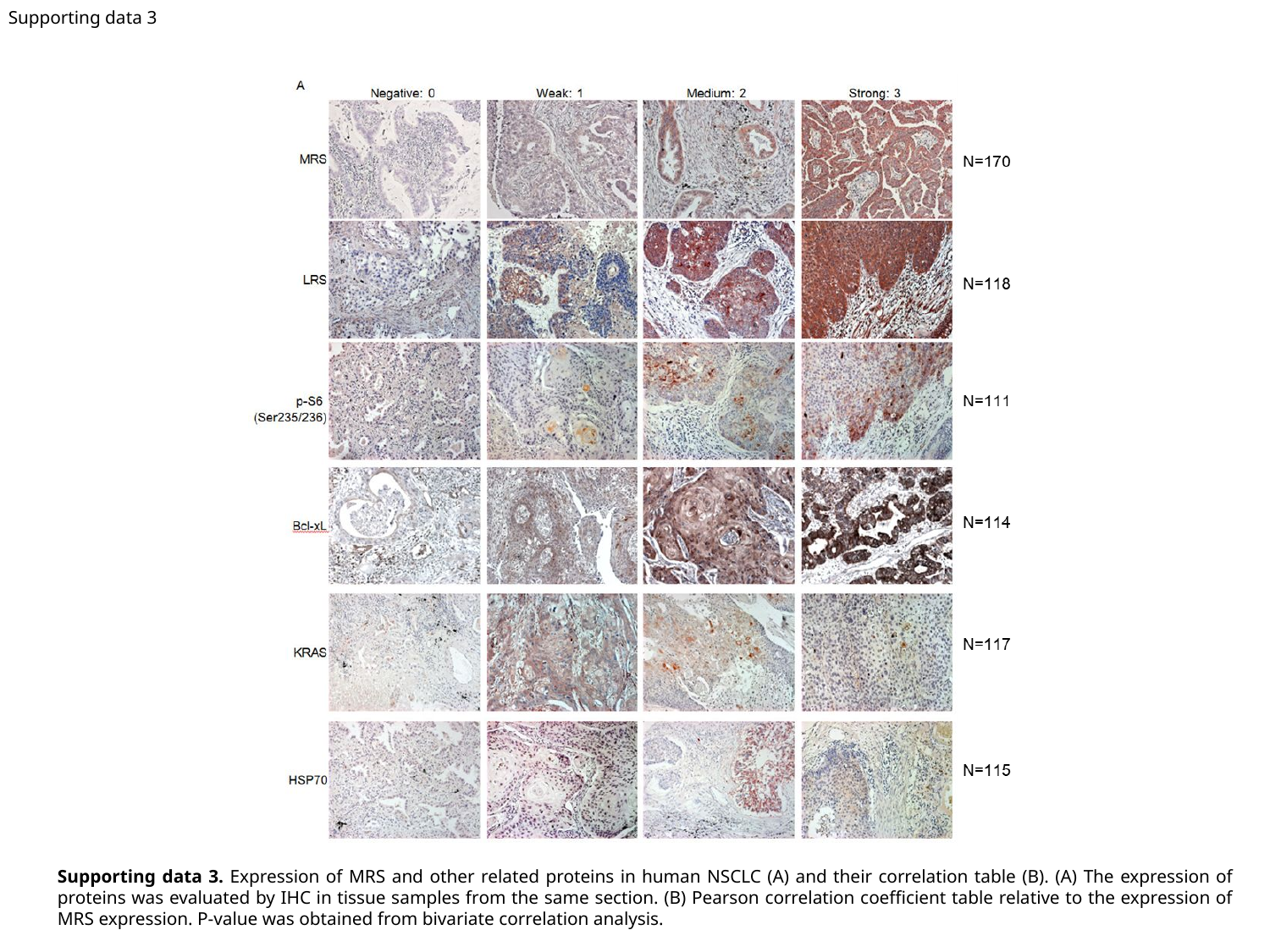

Supporting data 3
Supporting data 3. Expression of MRS and other related proteins in human NSCLC (A) and their correlation table (B). (A) The expression of proteins was evaluated by IHC in tissue samples from the same section. (B) Pearson correlation coefficient table relative to the expression of MRS expression. P-value was obtained from bivariate correlation analysis.

## Slide 2
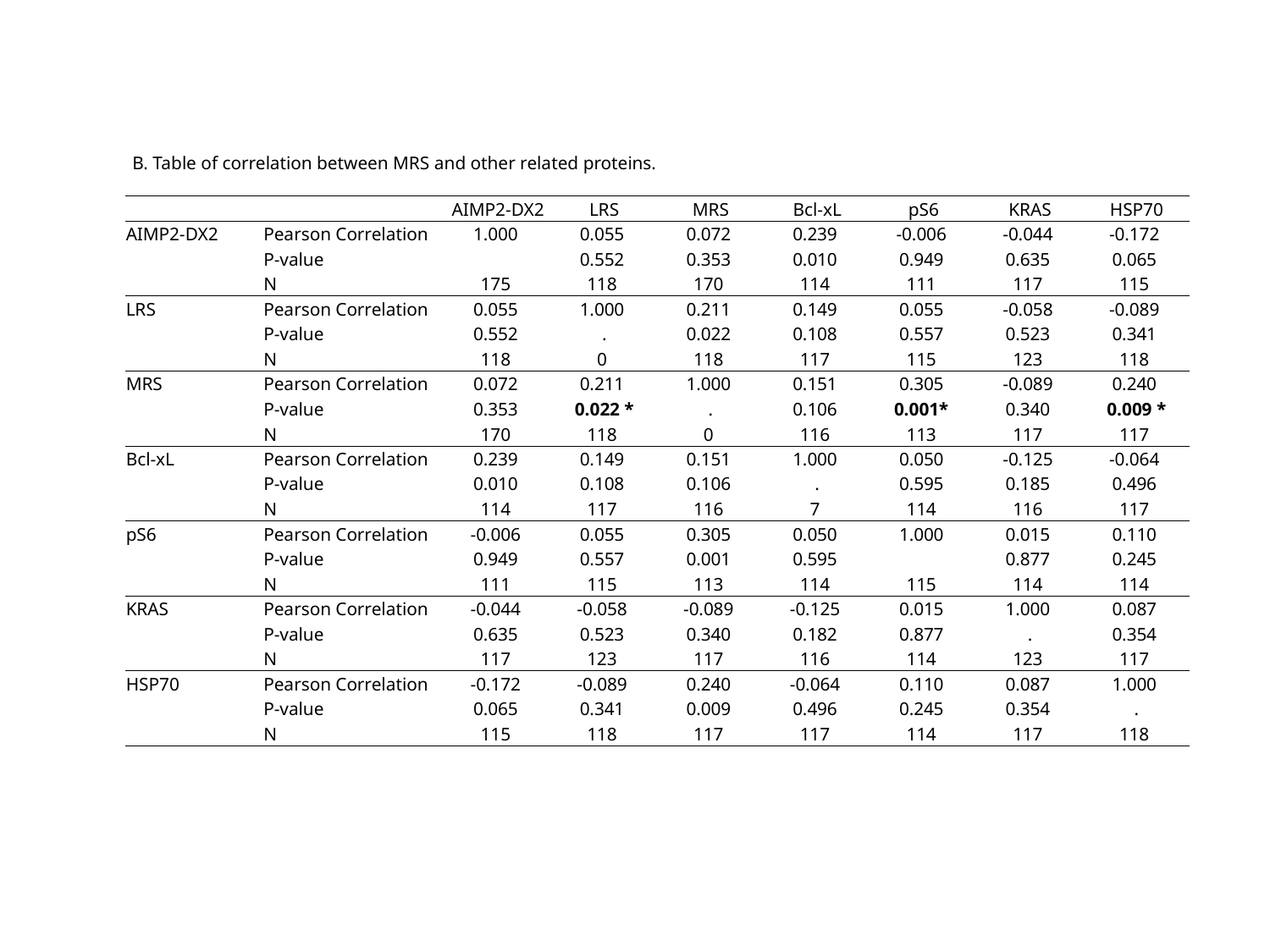

B. Table of correlation between MRS and other related proteins.
| | | AIMP2-DX2 | LRS | MRS | Bcl-xL | pS6 | KRAS | HSP70 |
| --- | --- | --- | --- | --- | --- | --- | --- | --- |
| AIMP2-DX2 | Pearson Correlation | 1.000 | 0.055 | 0.072 | 0.239 | -0.006 | -0.044 | -0.172 |
| | P-value | | 0.552 | 0.353 | 0.010 | 0.949 | 0.635 | 0.065 |
| | N | 175 | 118 | 170 | 114 | 111 | 117 | 115 |
| LRS | Pearson Correlation | 0.055 | 1.000 | 0.211 | 0.149 | 0.055 | -0.058 | -0.089 |
| | P-value | 0.552 | . | 0.022 | 0.108 | 0.557 | 0.523 | 0.341 |
| | N | 118 | 0 | 118 | 117 | 115 | 123 | 118 |
| MRS | Pearson Correlation | 0.072 | 0.211 | 1.000 | 0.151 | 0.305 | -0.089 | 0.240 |
| | P-value | 0.353 | 0.022 \* | . | 0.106 | 0.001\* | 0.340 | 0.009 \* |
| | N | 170 | 118 | 0 | 116 | 113 | 117 | 117 |
| Bcl-xL | Pearson Correlation | 0.239 | 0.149 | 0.151 | 1.000 | 0.050 | -0.125 | -0.064 |
| | P-value | 0.010 | 0.108 | 0.106 | . | 0.595 | 0.185 | 0.496 |
| | N | 114 | 117 | 116 | 7 | 114 | 116 | 117 |
| pS6 | Pearson Correlation | -0.006 | 0.055 | 0.305 | 0.050 | 1.000 | 0.015 | 0.110 |
| | P-value | 0.949 | 0.557 | 0.001 | 0.595 | | 0.877 | 0.245 |
| | N | 111 | 115 | 113 | 114 | 115 | 114 | 114 |
| KRAS | Pearson Correlation | -0.044 | -0.058 | -0.089 | -0.125 | 0.015 | 1.000 | 0.087 |
| | P-value | 0.635 | 0.523 | 0.340 | 0.182 | 0.877 | . | 0.354 |
| | N | 117 | 123 | 117 | 116 | 114 | 123 | 117 |
| HSP70 | Pearson Correlation | -0.172 | -0.089 | 0.240 | -0.064 | 0.110 | 0.087 | 1.000 |
| | P-value | 0.065 | 0.341 | 0.009 | 0.496 | 0.245 | 0.354 | . |
| | N | 115 | 118 | 117 | 117 | 114 | 117 | 118 |
