# Supplementary material for: Methionyl-tRNA synthetase overexpression is associated with poor clinical outcomes in non-small cell lung cancer
Source: BMC Cancer. 2017 Jul 5;17:467. doi: 10.1186/s12885-017-3452-9 (PMC5497355; doi:10.1186/s12885-017-3452-9)
Supplement: Supplementary file 4 — Supporting data 4.pptx Survival analysis according to the MRS level from TCGA NSCLC data set. Lung cancer cases of (A) all stages, (B) stage I ~ II, and (C) and III ~ IV were selected from lung adenocarcinoma and lung squamous cell carcinoma TCGA data set and DFS and OS was evaluated according to the MRS level. The expression of MRS was divided into two groups, upper 50% and lower 50%, based on the median value. P- values was obtained from Log-rank test. (PPTX 892 kb) [file 12885_2017_3452_MOESM4_ESM.pptx]

## Slide 1
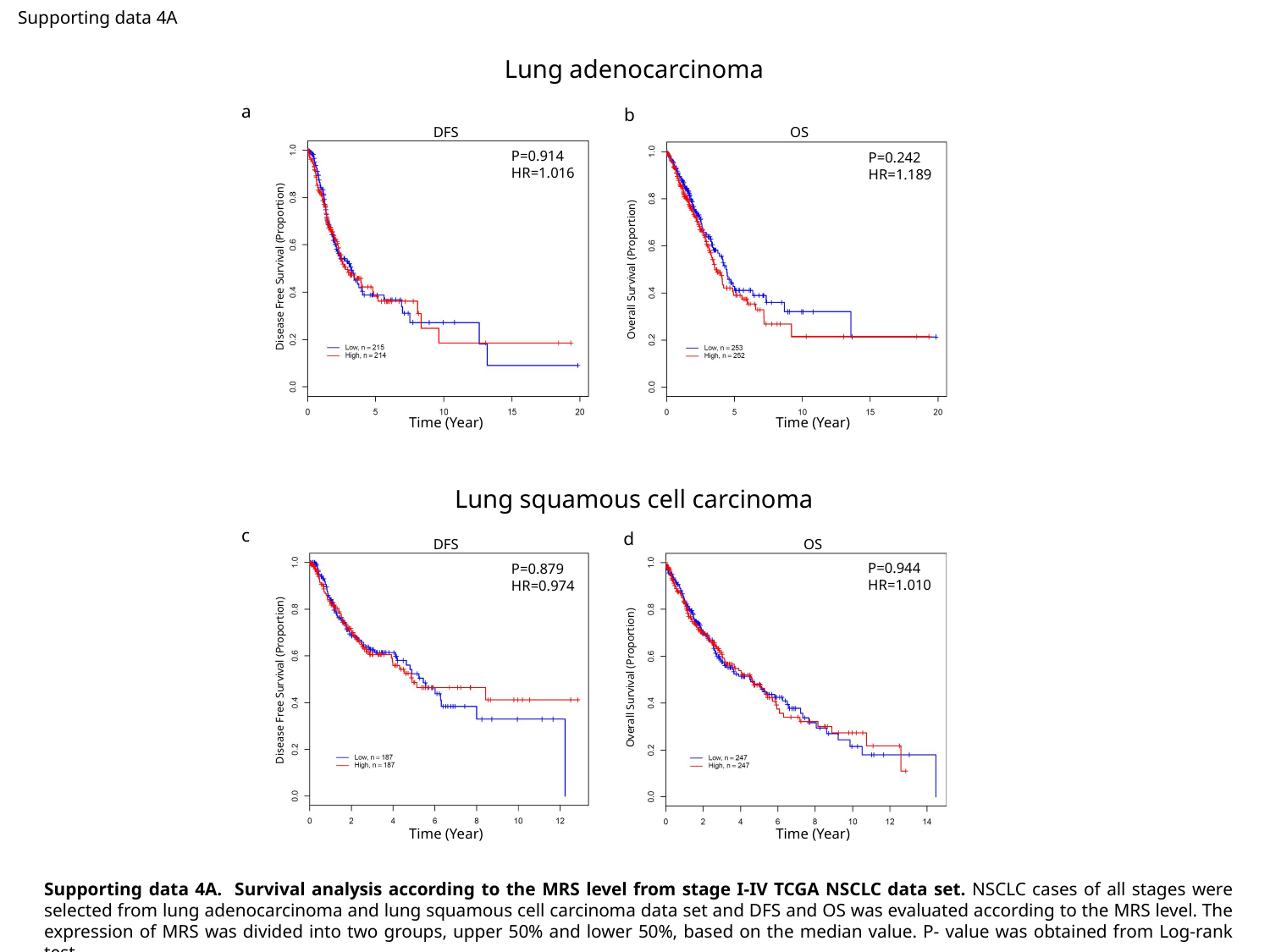

Supporting data 4A
Lung adenocarcinoma
a
b
DFS
OS
P=0.914
HR=1.016
P=0.242
HR=1.189
Disease Free Survival (Proportion)
Overall Survival (Proportion)
Time (Year)
Time (Year)
Lung squamous cell carcinoma
c
d
DFS
OS
P=0.944
HR=1.010
P=0.879
HR=0.974
Overall Survival (Proportion)
Disease Free Survival (Proportion)
Time (Year)
Time (Year)
Supporting data 4A. Survival analysis according to the MRS level from stage I-IV TCGA NSCLC data set. NSCLC cases of all stages were selected from lung adenocarcinoma and lung squamous cell carcinoma data set and DFS and OS was evaluated according to the MRS level. The expression of MRS was divided into two groups, upper 50% and lower 50%, based on the median value. P- value was obtained from Log-rank test.

## Slide 2
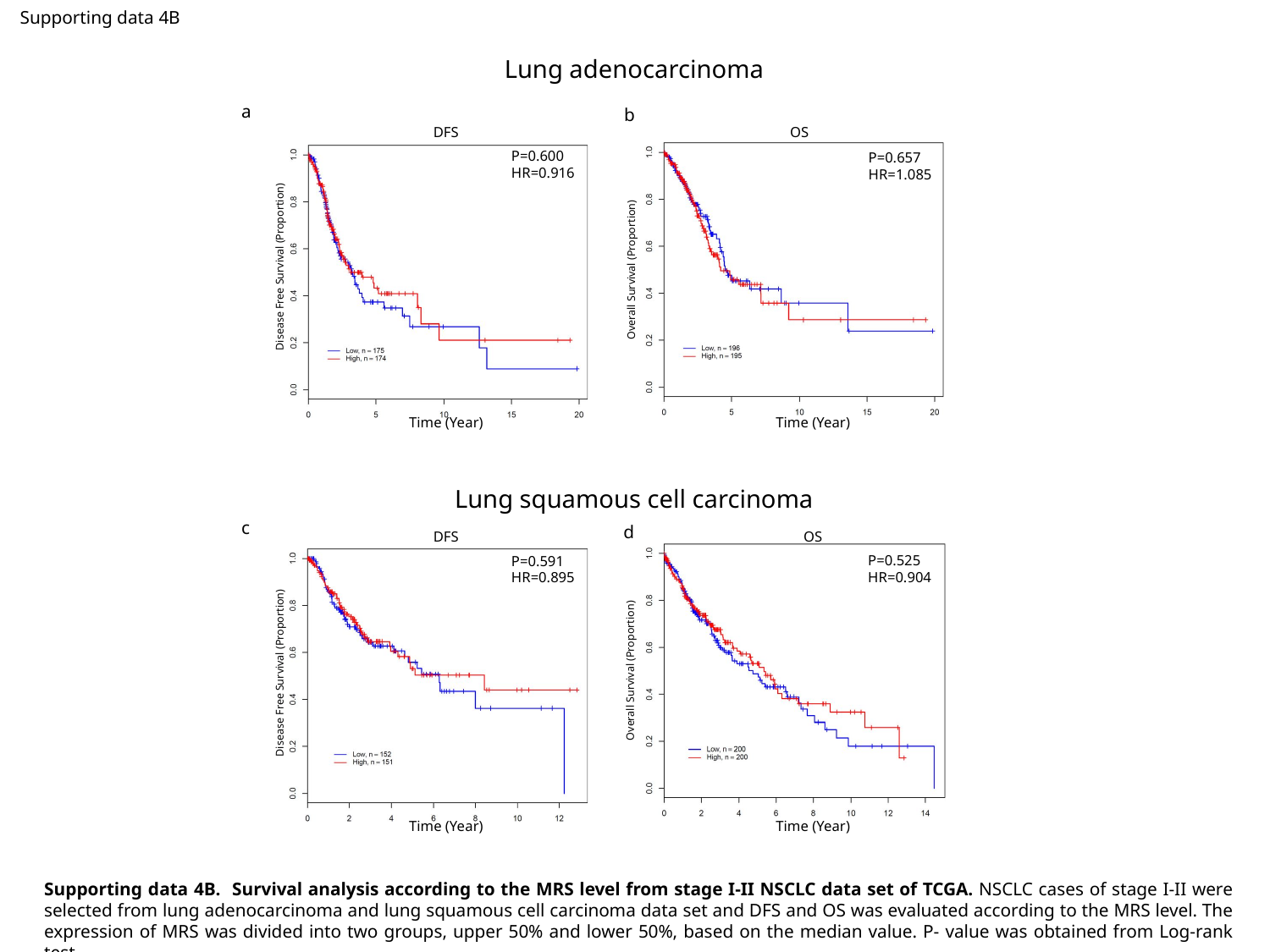

Supporting data 4B
Lung adenocarcinoma
a
b
DFS
OS
P=0.600
HR=0.916
P=0.657
HR=1.085
Disease Free Survival (Proportion)
Overall Survival (Proportion)
Time (Year)
Time (Year)
Lung squamous cell carcinoma
c
d
DFS
OS
P=0.525
HR=0.904
P=0.591
HR=0.895
Overall Survival (Proportion)
Disease Free Survival (Proportion)
Time (Year)
Time (Year)
Supporting data 4B. Survival analysis according to the MRS level from stage I-II NSCLC data set of TCGA. NSCLC cases of stage I-II were selected from lung adenocarcinoma and lung squamous cell carcinoma data set and DFS and OS was evaluated according to the MRS level. The expression of MRS was divided into two groups, upper 50% and lower 50%, based on the median value. P- value was obtained from Log-rank test.

## Slide 3
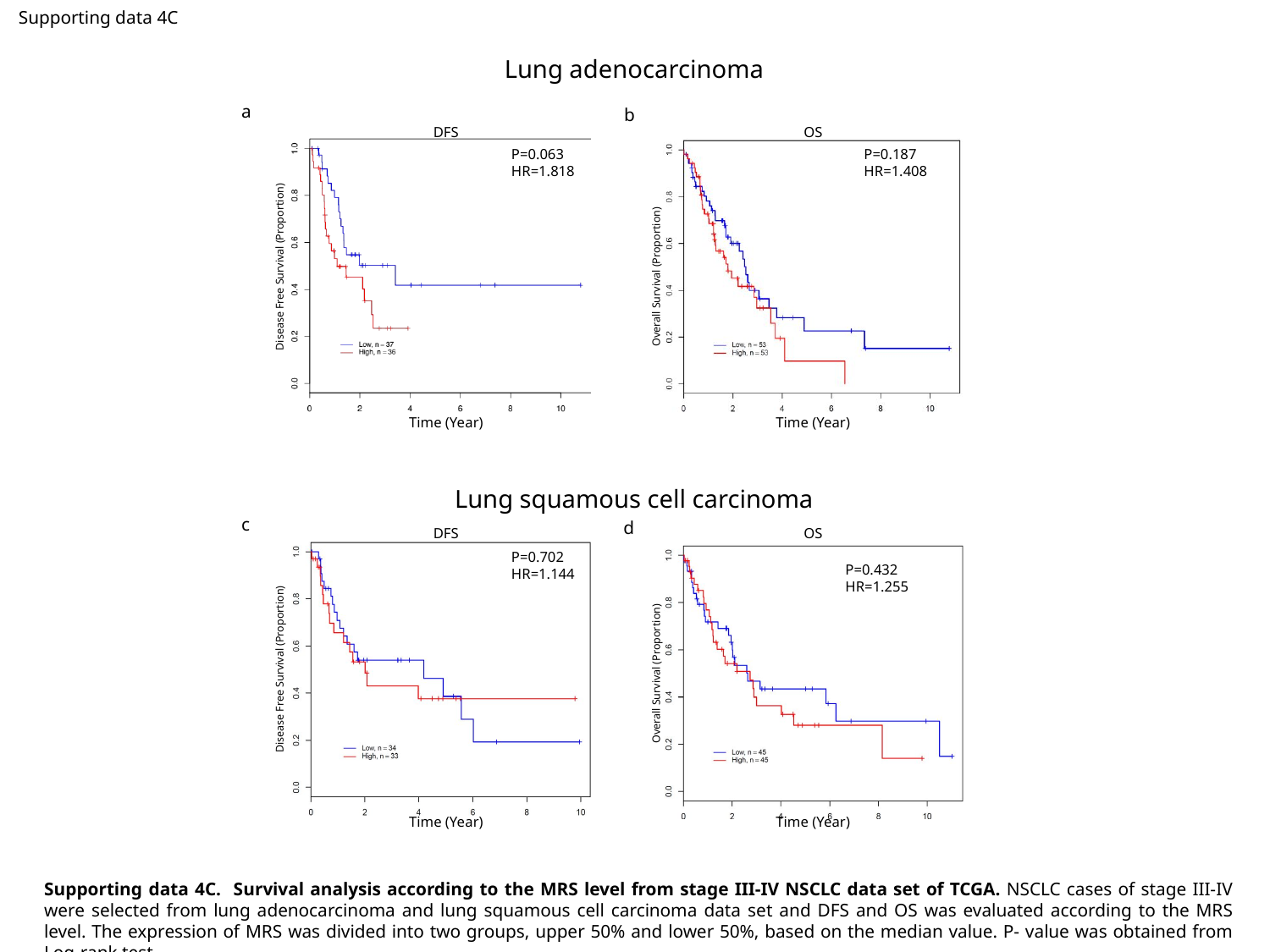

Supporting data 4C
Lung adenocarcinoma
a
b
DFS
OS
P=0.063
HR=1.818
P=0.187
HR=1.408
Disease Free Survival (Proportion)
Overall Survival (Proportion)
Time (Year)
Time (Year)
Lung squamous cell carcinoma
c
d
DFS
OS
P=0.702
HR=1.144
P=0.432
HR=1.255
Disease Free Survival (Proportion)
Overall Survival (Proportion)
Time (Year)
Time (Year)
Supporting data 4C. Survival analysis according to the MRS level from stage III-IV NSCLC data set of TCGA. NSCLC cases of stage III-IV were selected from lung adenocarcinoma and lung squamous cell carcinoma data set and DFS and OS was evaluated according to the MRS level. The expression of MRS was divided into two groups, upper 50% and lower 50%, based on the median value. P- value was obtained from Log-rank test.
